# Selvester Score May Be a Predictor of ICD Therapies in Patients with Non-Ischemic Dilated Cardiomyopathy

**DOI:** 10.21470/1678-9741-2021-0112

**Published:** 2022

**Authors:** Mevlut Serdar Kuyumcu, Mehmet Hakan Uzun, Yasin Ozen, Fatih Aksoy, Bayram Ali Uysal, Ercan Varol

**Affiliations:** 1 Department of Cardiology, Isparta Suleyman Demirel University, Faculty of Medicine, Isparta,Turkey.; 2 Department of Cardiology, Kahramanmaras Sutcu Imam University, Faculty of Medicine, Kahramanmaras, Turkey.; 3 Department of Cardiology, Ankara State Hospital, Ankara, Turkey.

**Keywords:** Cardiomyopathy, Dilated, Defibrillators, Implantable, Electrocardiography, Risk Factors, Sensitivity and Specificity, Survival Rate, Treatment Outcome

## Abstract

**Introduction:**

The benefit of implantable cardioverter-defibrillator (ICD) in patients with non-ischemic dilated cardiomyopathy (DCM) is still an issue under discussion. Studies examining the relationship between ventricular scar tissue and ICD shock with cardiac magnetic resonance (CMR) are promising. CMR studies have shown that ventricular scar tissue size and Selvester score show a correlation. In the light of this information, this study aimed to investigate the potential relationship between Selvester score and ICD therapies.

**Methods:**

The study included 48 patients who had undergone ICD implantation with a diagnosis of DCM and who had undergone routine 6-month ICD control in outpatient clinic controls between December 2018 and October 2019. Selvester score and other data were compared between patients who received ICD therapy (n=10) and those who did not (n=38).

**Results:**

Selvester score (P<0.001) was higher in ICD therapy group. Positive correlation was found between ICD shock therapy and Selvester score (P=0.002, r=0.843). Selvester score was detected as an independent predictor for ICD therapy after multiple linear regression analysis (P=0.004). Receiver operating characteristic curve analysis showed that Selvester score (P<0.001) was a significant predictor of ICD therapy. Selvester score cutoff points of 5 for were calculated to estimate ICD therapy, with a sensitivity of 100% and specifity of 81%.

**Conclusion:**

In our study, it was found that a high Selvester score may be a predictor for ICD therapies in patients with DCM. As an inexpensive and non-invasive method, Selvester score can help in the decision-making in these patients.

**Table:** Abbreviations, Acronyms & Symbols

ATP	= Anti-tachycardia pacing
AUC	= Area under the curve
CI	= Cardiac index
CMR	= Cardiac magnetic resonance
DCM	= Dilated cardiomyopathy
ECG	= Electrocardiogram
ICD	= Implantable cardioverter-defibrillator
LVEF	= Left ventricular ejection fraction
OR	= Odds ratio
SCD	= Sudden cardiac death
SPSS	= Statistical Package for the Social Sciences

## INTRODUCTION

Life-threatening ventricular arrhythmias, including sustained ventricular tachycardia and ventricular fibrillation, are common in patients with systolic heart failure and non-ischemic dilated cardiomyopathy (DCM), which may lead to sudden cardiac death (SCD)^[1]^. Primary prevention of SCD refers to medical or interventional therapy undertaken to prevent SCD in patients who have not experienced symptomatic life-threatening sustained ventricular tachycardia and ventricular fibrillation or sudden cardiac arrest, but who are felt to be at an increased risk for such an event^[2]^. Primary prevention of SCD in patients with heart failure and cardiomyopathy with reduced left ventricular ejection fraction (LVEF), either due to coronary heart disease or a dilated non-ischemic etiology, will be reviewed here with an emphasis on the role of implantable cardioverter-defibrillators (ICD)^[1]^. However, the benefit of ICD in patients with dilated cardiomyopathy is still an issue under discussion^[3]^. So far, there have been no randomized trials of ICD *versus* control group that reported a reduction in all-cause mortality in patients with DCM. Only a subgroup analysis of the Sudden Cardiac Death in Heart Failure Trial (SCD-HeFT) showed a trend towards reduced mortality in these patients^[4]^. Recently, the Danish Study to Assess the Efficacy of ICDs in Patients with Non-ischemic Systolic Heart Failure on Mortality (DANISH) trial showed no reduction in the primary endpoint of all-cause mortality among ICD recipients^[5]^. ICD implantation had only a reducing effect on SCD.

The 12-lead electrocardiogram (ECG) is a low-cost, noninvasive, reproducible, rapid, standard cardiac examination method that is used anywhere. Abnormal findings on ECG such as fragmented QRS or bundle branch block and prolonged QRS duration were reported as prognostic predictors in heart failure patients. In the 1980s, Selvester et al.^[6]^ developed a unique QRS scoring system composed of 32 points, in which each point was allocated 3% of the left ventricular mass. In addition, the Selvester score was found to be a predictor of mortality and morbidity in DCM patients^[7,8]^. Cardiac magnetic resonance (CMR) studies show that ventricular scar tissue size and Selvester score have a good correlation^[6,9]^.

Studies examining the relationship between ventricular scar tissue and ICD shock with CMR are promising^[10,11]^. However, development of a simple, low-cost and noninvasive method for risk stratification is urgently required to reduce healthcare costs and reduce the burden of heart failure for patients and medical staff^[3]^. In the light of this informations, we aimed to investigate the potential relationship between Selvester score and ICD therapies.

## METHODS

The study evaluated 51 patients who had undergone ICD implantation for primary prevention of sudden cardiac death (heart failure with LVEF ≤30% or LVEF ≤35 with New York Heart Association functional classification II-IV)^[12]^ with a diagnosis of DCM (non-ischemic etiology, confirmed by either invasive coronary angiography or coronary computed tomographic angiography)^[3]^ and who had undergone 6-month routine ICD control in outpatient clinic visits between December 2018 and October 2019. ECG performed during hospitalization for the purpose of ICD implantation of the patients was used in our study. A total of 3 patients did not come to the 6^th^ month assessment. These patients were excluded from the study. A total of 48 patients were included in the study. Selvester score and other data were compared between patients who received ICD therapy [shock and anti-tachycardia pacing (ATP)], [ICD therapy (+), n=10] and those who did not receive ICD theraphy [ICD therapy (-), n=38].

The severity of the heart failure symptoms was assessed using the New York Heart Association (NYHA) functional classification. Electronic medical records were used to obtain patients’ medical histories. The diagnosis of hypertension was made when the systolic blood pressure was 140 mmHg or higher, if the diastolic blood pressure was 90 mmHg or higher by at least three different measurements, or if the patient used anti-hypertensive medication. The diagnosis of diabetes mellitus was established by a fasting blood glucose of 126 mg/dL or higher, or with the use of antidiabetic medication. All patients provided written informed consent and the study protocol was approved by the local Ethics Committee. The study was conducted in accordance with the Declaration of Helsinki, Good Clinical Practice and International Conference on Harmonisation guidelines.

### Echocardiography

Echocardiography was performed in all patients when the decision for ICD implantation was made in the outpatient clinic. Echocardiographic assessment was performed using an iE33 xMATRIX Cardiovascular Ultrasound System (Koninklijke Philips N.V. Amsterdam, Netherlands) with a 3.5 MHz transducer. The echocardiographic examination was performed in the left lateral position. Parasternal long- and short-axis views and apical views were used as standard imaging windows. LVEF was calculated by using a modified Simpson method. All echocardiographic images were analyzed by an experienced cardiologist.

### Electrocardiography and Selvester Score Evaluation

**Fig. 1:**
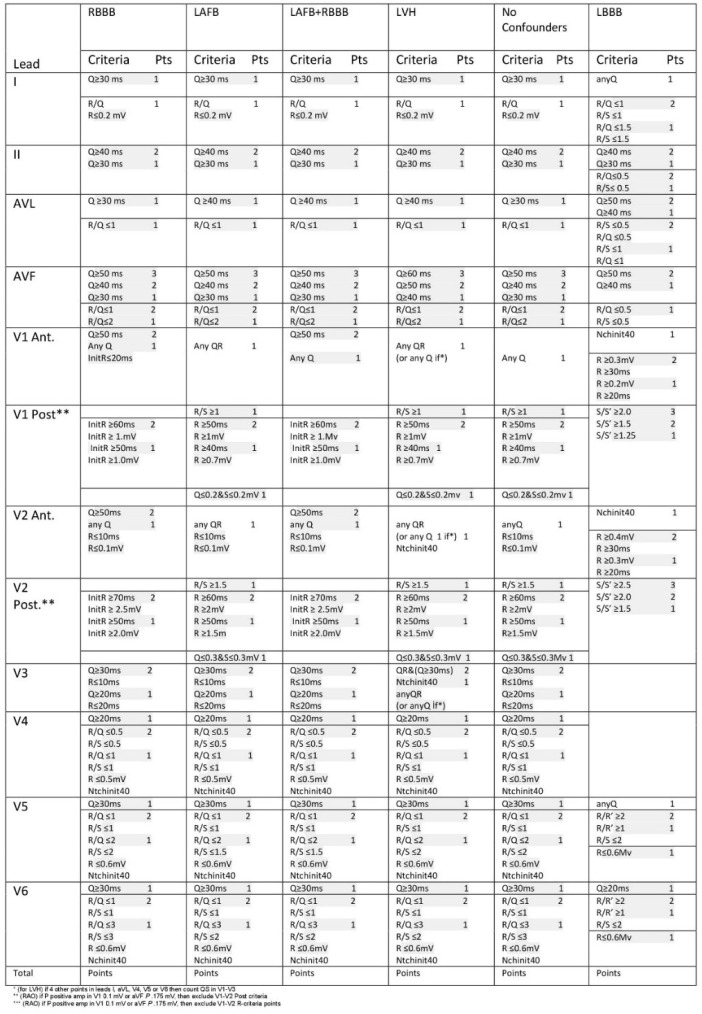
Selvester score chart.

Prior to hospitalization, 12-lead ECG records were taken using an electrocardiograph (FCP-7541; Fukuda Denshi Co. Ltd, Tokyo, Japan). The 12-lead ECG was recorded at a paper speed of 50 mm/s in the supine position. All ECGs were scanned and transferred to a personal computer to decrease measurement errors, and then 400% magnification by Adobe Photoshop software was used. An average value of three readings was calculated for each lead. An experienced cardiologist, who were blinded to other patient information, manually calculated the 32-point Selvester QRS score, based on an algorithm reported in the literature (Figure 1)^[9]^.

### Implantable Cardioverter-Defibrillator Implantation and Follow-Up

Single-chamber, dual-chamber or cardiac resynchronization therapy devices with defibrillator capability were implanted through standard techniques by experienced operators, following guideline recommendations^[13]^. After implantation, pneumothorax was detected in 1 patient in ICD therapy (-) group; pneumothorax was not detected in ICD therapy (+) group. Pocket hematoma was detected in 2 patients in ICD therapy (-) group and in 1 patient in ICD therapy (+) group (Table 1). In all patients, ATP algorithms were also included. Detection of duration criteria was programmed to require the tachycardia to continue for at least 6-12 seconds or for at least 30 intervals before completing detection. Supraventricular/ventricular tachycardia discriminators were activated according to manufacturer’s software specifications. Device shocks were evaluated according to guidelines^[14]^. First ICD controls were made 30 days after implantation. Eventual evaluations of the devices that were made 180 days after implantation were taken into consideration in the study. Inappropriate ICD shocks and ATP therapies were not included in the evaluations. 2 patients in ICD therapy (+) group and 3 patients in ICD therapy (-) group were hospitalized due to decompensated heart failure (Table 1). No mortality was detected during the follow-up period in both groups.

**Table 1:** ECG and ICD parameters of the patients.

Variables	ICD therapy (+)	ICD therapy (-)	*P*-value
	(n=10)	(n=38)	
Heart rate, bpm	72.5±8.9	73.5±13.3	0.815
QRS duration, ms	65.3±8.6	61.2±10.7	0.251
Corrected QT duration, ms	418±29	396±22	**0.018**
CRT device, n (%)	2 (20.0)	6 (15.8)	0.751
VT 1 zone, ms	340±20	335±60	0.826
VT 2 zone, ms	305±20	303±42	0.831
VF zone, ms	292±9	293±16	0.807
Monitored non-sustained VT episodes	10.6±2	1.6±1	**<0.001**
ATP therapies	1		
Shock	3		
Shock after ATP	2		
Shock and ATP in different episodes	4		
Selvester score	6.3±1.8	4.1±2.4	**<0.001**
Sinus rhythm	6 (60.0%)	20 (52.6%)	0.677
Left bundle branch block	2 (20.0)	6 (15.8)	0.751
Left anterior fascicular block	1 (10.0%)	3 (7.9%)	0.709
Left posterior fascicular block	0 (0.0%)	2 (5.3%)	0.459
Right bundle branch block	1 (10.0%)	6 (15.8)	0.644
Right bundle branch block + left anterior fascicular block	0 (0.0%)	1 (2.6%)	0.604

Data are given as mean±standart deviation, number or median (interquartile range). ATP=antitachycardia pacing; CRT=cardiac resynchronization therapy; VF=ventricular fibrillation; VT=ventricular tachycardia

### Statistical Analysis

Using SPSS for Windows version 21.0 (SPSS, Chicago, IL, USA), the mean, standard deviation, rate and frequency values were used for the statistical analyses. The sample size of each group was adjusted for more than 10 patients because we calculated the minimum number of individuals that should be sampled with 90% power and 0.05 type I error as at least 46 (R 3.0.1. open source program). The primary effect variable was determined as one point of Selvester score chart. The normal distribution of continuous variables was assessed using Kolmogorov-Smirnov test. Parametric data were analyzed with Student’s t-test, and non-parametric data were analyzed with Mann-Whitney U test. Intergroup comparative analysis was carried out using the chi-square test for categorical variables. Logistic regression model was established to explain the linearity between relevant variables. We used standardized beta coefficients and 95% confidence intervals, and statistical significance was accepted as a *P*-value <0.05. The optimal cutoff value of systemic immune-inflammation index (SII) to predict ICD therapies was assessed by calculating the area under the curve of the receiver operating characteristic curve. The Youden index was used to determine cutoff values.

## RESULTS

Demographic, echocardiographic and drug use characteristics of patients are shown in Table 2. There was no difference between groups, except for LVEF (*P*=0.019). Electrocardiogram and ICD parameters of the patients are shown in Table 1. Corrected QT duration was longer in ICD therapy (+) group (*P*=0.018). Monitored non-sustained ventricular tachycardia episodes were higher in ICD therapy (+) group (*P*<0.001). Mean Selvester score was higher in ICD therapy (+) group (*P*<0.001).

**Table 2:** Demographic, echocardiographic and drug use characteristics of patients.

Variables	ICD therapy (+)	ICD therapy (-)	*P*-value
	(n=10)	(n=38)	
Age, years	65.3±8.6	61.2±10.7	0.251
Female, n (%)	1 (10.0)	8 (21.1)	0.426
Hypertension, n (%)	3 (30.0)	10 (26.3)	0.859
Diabetes mellitus, n (%)	2 (20.0)	9 (23.7)	0.805
EF, (%)	26.3±4.6	29.4±7.2	**0.019**
Mean NYHA score	2.00±0.9	1.82±0.8	0.522
Usage of beta-blockers, n (%)	10 (100.0)	34 (89.5)	0.284
Usage of ACEi/ARB, n (%)	7 (70.0)	30 (78.9)	0.549
Usage of sacubitril-valsartan, n (%)	1 (10.0)	2 (5.3)	0.582
Usage of mineralocorticoid antagonist, n (%)	6 (60.0)	24 (63.2)	0.854
Usage of diuretics, n (%)	5 (50.0)	23 (60.5)	0.548
Usage of digoxin, n (%)	2 (20.0)	10 (26.3)	0.682
Usage of ivabradine, n (%)	1 (10.0)	4 (10.5)	0.961
Usage of amiodarone, n (%)	1 (10.0)	2 (5.3)	0.582
Usage of mexiletine, n (%)	0 (0.0)	0 (0.0)	0.403
Usage of other antiarrhythmics, n (%)	0 (0.0)	0 (0.0)	

Data are given as mean±standart deviation, number or median (interquartile range). ACEi/ARB=angiotensin-converting enzyme inhibitors and angiotensin receptor blockers; EF=ejection fraction; NYHA=New York Heart Association

The predictors (Tables 1 and 2) of ICD therapy were determined through univariate and multiple linear regression analyzes, and the results are shown in Table 3. In the univariate regression analysis, higher Selvester score [odds ratio (OR)=0.320; 95% confidence interval (CI): 0.144-0.709; *P*=0.002], longer corrected QT duration (OR=0.971; 95% CI: 0.945-0.997; *P*=0.029), and lower LVEF (OR=1.077; 95% CI: 0.944-1.227; *P*=0.047) were associated with ICD therapy. Multiple linear regression analysis demonstrated that higher Selvester score (OR=1.068; 95% CI: 1.017-1.122; *P*=0.008) was an independent predictor of ICD therapy.

**Table 3:** Multivariate logistic regression analysis showing predictors for ICD therapies.

	Univariable		Multivariable	
Variables	Beta (95% CI)	*P*-value	Beta (95% CI)	*P*-value
Selvester score	0.320 (0.144-0.709)	**0.002**	0.328 (0.134-0.799)	**0.004**
Corrected QT duration	0.971 (0.945-0.997)	**0.029**	0.975 (0.946-1.006)	0.108
EF	1.077 (0.944-1.227)	**0.047**	0.995 (0.839-1.180)	0.217

EF=ejection fraction

**Fig. 2:**
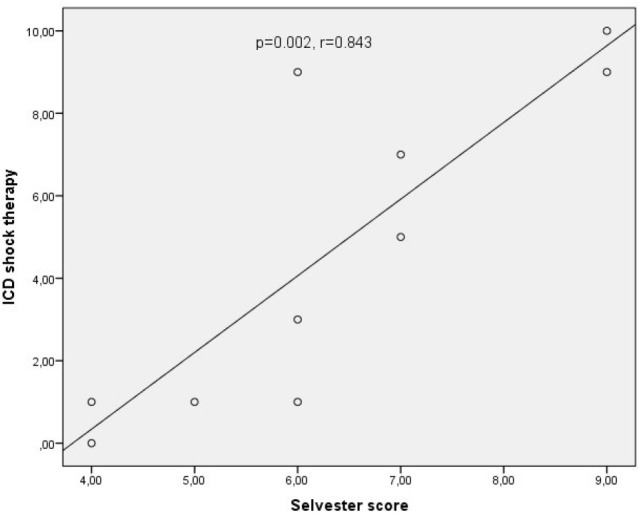
Correlation between Selvester score and ICD shock therapies. ICD=implantable cardioverter-defibrillator

In correlation analysis, a positive correlation was found between ICD shock therapy and the Selvester score (*P*=0.002, r=0.843), (Figure 2).

**Fig. 3:**
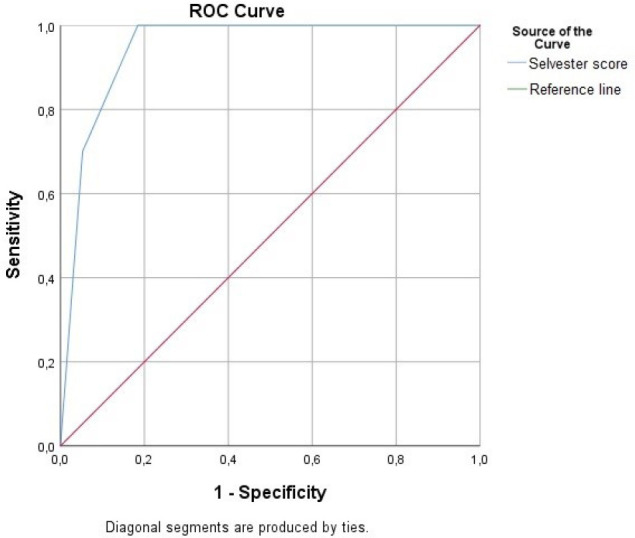
Receiver operating characteristic curve analysis of Selvester score.

Analysis of the receiver operating characteristic curve showed that Selvester score (AUC: 0.946; 95% CI: 0.841-0.997; *P*<0.001) was a significant predictor of ICD therapy. The cutoff points of 5 for Selvester score were calculated to estimate ICD therapy, with a sensitivity of 100% and specifity of 81% (Figure 3).

## DISCUSSION

In our study, the Selvester score was found to be significantly higher in ICD therapy (+) group, and a significant correlation was found between the ICD shock rate and the Selvester score. For the first time in the literature, it has been determined that the Selvester score can be a predictor of ICD shock and a cutoff value that can be used to predict ICD shocks in routine clinical practice has been determined. In our study, the mean LVEF was found to be lower in the ICD shock group. Although low LVEF is the most important predictor of SCD, it is one of the most important predictors of arrhythmic substrate due to cardiac fibrosis in the myocardium, supporting our hypothesis^[1]^.Also in our study, corrected QT distance was found to be long in ICD therapy (+) group, and long QT duration is associated with an increase in SCD and arrythmogenic events. QT interval prolongation may be associated with drug side effects and congenital diseases, as well as direct damage to the conduction system and fibrosis^[15]^. In another finding from our study, non-sustained attacks were found to be higher in the ICD shock group, which is associated with an increase in the incidence of SCD^[16]^.

DCM is a disease seen with a prevalence of 1/2,500 in the general population. The annual incidence of SCD is around 2-4% in patients with DCM. Remarkably, half of all causes of death in these patients are SCD. In a study examining patients who survived cardiac arrest, DCM was found to be the responsible disease between 10-19% of patients^[17]^. In a study examining patients who survived cardiac arrest, DCM was found to be the responsible disease in 10-19% of the cases^[18]^. More importantly, SCD may be the first manifestation of DCM in previously asymptomatic individuals. However, no difference in mortality has yet been reported among DCM patients with and without ICD implantation in any randomized trial. A subgroup analysis of the SCD-HeFT trial had only reported a trend towards reduced mortality in DCM^[4]^.

In the multicenter DANISH study, conducted in Denmark, the benefit of the ICD implanted for primary prevention in non-ischemic systolic heart failure was investigated. Patients with NT-proBNP >200 pg/mL, NYHA class II-IV and LVEF ≤35% were randomly assigned to the ICD group or the control group. While the primary endpoint of the study was death from any cause, the secondary endpoints were sudden cardiac death and cardiovascular death. As a result of the study, ICD for primary prevention was not found to be beneficial in non-ischemic systolic heart failure. However, ICD implantation had a positive effect on SCD incidents^[5]^.

In a selected group of DCM patients, a potential mortality benefit was found with ICD implantation. However, algorithms are needed to facilitate the selection of these patients and reduce unnecessary ICD implantations.

One of the mechanisms of myocardial fibrosis is due to collagen deposition that causes interstitial expansion (interstitial fibrosis). Furthermore, increase in collagen tissue with replacement fibrosis may occur due to progressive cardiomyocyte death^[19]^. Myocardial fibrosis is a substrate for ventricular arrhythmias in ischemic heart disease, in which the scar represents a transition point between normal myocardium and fibrotic tissue and causes slow conduction re-entry circuits thus results in ‘scar-associated’ ventricular tachycardia^[20]^.

CMR provides a reproducible assessment of LVEF and left ventricular volumes, and the application of gadolinium contrast provides data on myocardial scarring. In a large meta-analysis of 2,948 DCM patients, a strong association between myocardial fibrosis shown by late gadolinium enhancement images and SCD due to arrhythmia was found^[21]^. It was determined in a study that patients with LVEF >30% and late gadolinium enhancement involving >5% of their left ventricular mass were as likely to die or receive an ICD shock for ventricular tachycardia as those with LVEF <30%. Conversely, patients with LVEF <30% who had minimal or no late gadolinium enhancement did just as well as those patients with LVEF <30%. The ability of late gadolinium enhancement burden to risk stratify patients was independent of LVEF^[22]^. In addition, lack of late gadolinium enhancement is associated with reverse remodelling in DCM, suggesting that ICD implantation may be safely delayed even after 3 months of optimal medical therapy in selected patients, to wait for a significant geometrical and functional recovery^[23]^.

Selvester QRS scoring system is a system calculated over a 12-lead standard ECG and it has been shown that every 1-point increase in the score is associated with a 1.32-fold increase in the risk of a cardiac event^[24,25]^. Basically, with this scoring system, myocardial scar volume is quantitatively calculated^[26]^. It was shown that the QRS scoring system can be used with all types of ventricular conduction types, and can be applied to patients with both ischemia and DCM^[27]^. Moreover, Rosengarten et al.^[28]^ reported that QRS scoring was very useful to quantify transmural scar, and showed an association with medium-term mortality risk. In another study, a strong correlation was found between the Selvester Score and the scar burden detected by CMR. In this study, it was found that the increased Selvester score was closely related to the incidence of ICD therapies, caused by arrhythmic events related to the possible ventricular scar burden^[29]^.

### Limitations of the Study

The present study is a cross-sectional study with a relatively small sample size. Data on major adverse cardiovascular events during the follow-up was not available for the patient population studied. Patients were not scanned and correlated with CMR, which is a more specific method for scar screening. Therefore, the results of the present study should be verified in multicenter prospective longitudinal studies in a larger sample size. The limitations of this study should be considered when interpreting the results.

## CONCLUSION

In the present study, the Selvester score was found to be a predictor for arrhythmic events in DCM patients. Which patients with DCM are most likely to benefit from ICD therapy is an active topic of debate. Our study has the potential to guide the determination of ICD indications in DCM patients. However, further studies are needed to better establish the relationship between arrhythmic events in DCM and Selvester score.

## References

[1] Saour B, Smith B, Yancy CW (2017). Heart failure and sudden cardiac death. Card Electrophysiol Clin.

[2] Hindricks G, Lenarczyk R, Kalarus Z, Döring M, Shamloo AS, Dagres N (2018). Prevention of sudden cardiac death by the implantable
cardioverter-defibrillator. Pol Arch Intern Med.

[3] Akhtar M, Elliott PM (2019). Risk stratification for sudden cardiac death in non-ischaemic
dilated cardiomyopathy. Curr Cardiol Rep.

[4] Petrie MC, Connelly DT, Gardner RS (2018). Who needs an implantable cardioverter-defibrillator?
Controversies and opportunities after DANISH. Eur J Heart.

[5] Køber L, Thune JJ, Nielsen JC, Haarbo J, Videbæk L, Korup E (2016). Defibrillator implantation in patients with nonischemic systolic
heart failure. N Engl J Med.

[6] Selvester RH, Wagner GS, Hindman NB (1985). The selvester QRS scoring system for estimating myocardial
infarct size. The development and application of the system. Arch Intern Med.

[7] Hiraiwa H, Okumura T, Sawamura A, Sugiura Y, Kondo T, Watanabe N (2018). The Selvester QRS score as a predictor of cardiac events in
nonischemic dilated cardiomyopathy. J Cardiol.

[8] Hiraiwa H, Okumura T, Sawamura A, Murohara T (2018). Author's reply. J Cardiol.

[9] Strauss DG, Selvester RH (2009). The QRS complex--a biomarker that "images" the heart: QRS scores
to quantify myocardial scar in the presence of normal and abnormal
ventricular conduction. J Electrocardiol.

[10] Memon S, Ganga HV, Kluger J (2016). Late gadolinium enhancement in patients with nonischemic dilated
cardiomyopathy. Pacing Clin Electrophysiol.

[11] Strauss DG, Poole JE, Wagner GS, Selvester RH, Miller JM, Anderson J (2011). An ECG index of myocardial scar enhances prediction of
defibrillator shocks: an analysis of the sudden cardiac death in heart
failure Trial. Heart Rhythm.

[12] Ponikowski P, Voors AA, Anker SD, Bueno H, Cleland JG, Coats AJ (2016). 2016 ESC guidelines for the diagnosis and treatment of acute and
chronic heart failure: the task force for the diagnosis and treatment of
acute and chronic heart failure of the European society of cardiology
(ESC). Developed with the special contribution of the heart failure association
(HFA) of the ESC. Eur J Heart Fail.

[13] Ponikowski P, Voors AA, Anker SD, Bueno H, Cleland JGF, Coats AJS (2016). 2016 ESC guidelines for the diagnosis and treatment of acute and
chronic heart failure: the task force for the diagnosis and treatment of
acute and chronic heart failure of the European society of cardiology
(ESC)Developed with the special contribution of the heart failure
association (HFA) of the ESC. Eur Heart J.

[14] Wilkoff BL, Fauchier L, Stiles MK, Morillo CA, Al-Khatib SM, Almendral J (2016). 2015 HRS/EHRA/APHRS/SOLAECE expert consensus statement on optimal
implantable cardioverter-defibrillator programming and
testing. Heart Rhythm.

[15] O'Neal WT, Singleton MJ, Roberts JD, Tereshchenko LG, Sotoodehnia N, Chen LY (2017). Association between QT-interval components and sudden cardiac
death: the ARIC study (atherosclerosis risk in communities). Circ Arrhythm Electrophysiol.

[16] de Sousa MR, Morillo CA, Rabelo FT, Nogueira Filho AM, Ribeiro AL (2008). Non-sustained ventricular tachycardia as a predictor of sudden
cardiac death in patients with left ventricular dysfunction: a
meta-analysis. Eur J Heart Fail.

[17] Kadish A, Dyer A, Daubert JP, Quigg R, Estes NA, Anderson KP (2004). Prophylactic defibrillator implantation in patients with
nonischemic dilated cardiomyopathy. N Engl J Med.

[18] Deo R, Albert CM (2012). Epidemiology and genetics of sudden cardiac death. Circulation.

[19] Beltrami CA, Finato N, Rocco M, Feruglio GA, Puricelli C, Cigola E (1995). The cellular basis of dilated cardiomyopathy in
humans. J Mol Cell Cardiol.

[20] McCrohon JA, Moon JC, Prasad SK, McKenna WJ, Lorenz CH, Coats AJ (2003). Differentiation of heart failure related to dilated
cardiomyopathy and coronary artery disease using gadolinium-enhanced
cardiovascular magnetic resonance. Circulation.

[21] Di Marco A, Anguera I, Schmitt M, Klem I, Neilan TG, White JA (2017). Late gadolinium enhancement and the risk for ventricular
arrhythmias or sudden death in dilated cardiomyopathy: systematic review and
meta-analysis. JACC Heart Fail.

[22] Klem I, Weinsaft JW, Bahnson TD, Hegland D, Kim HW, Hayes B (2012). Assessment of myocardial scarring improves risk stratification in
patients evaluated for cardiac defibrillator implantation. J Am Coll Cardiol.

[23] Barison A, Aimo A, Ortalda A, Todiere G, Grigoratos C, Passino C (2018). Late gadolinium enhancement as a predictor of functional
recovery, need for defibrillator implantation and prognosis in non-ischemic
dilated cardiomyopathy. Int J Cardiol.

[24] Sobue Y, Harada M, Koshikawa M, Ichikawa T, Yamamoto M, Okuda K (2015). QRS-based assessment of myocardial damage and adverse events
associated with cardiac sarcoidosis. Heart Rhythm.

[25] Wieslander B, Nijveldt R, Klem I, Lokhnygina Y, Pura J, Wagner GS (2015). Evaluation of selvester QRS score for use in presence of
conduction abnormalities in a broad population. Am Heart J.

[26] Loring Z, Chelliah S, Selvester RH, Wagner G, Strauss DG (2011). A detailed guide for quantification of myocardial scar with the
selvester QRS score in the presence of electrocardiogram
confounders. J Electrocardiol.

[27] Strauss DG, Selvester RH, Lima JA, Arheden H, Miller JM, Gerstenblith G (2008). ECG quantification of myocardial scar in cardiomyopathy patients
with or without conduction defects: correlation with cardiac magnetic
resonance and arrhythmogenesis. Circ Arrhythm Electrophysiol.

[28] Rosengarten JA, Scott PA, Chiu OK, Shambrook JS, Curzen NP, Morgan JM (2013). Can QRS scoring predict left ventricular scar and clinical
outcomes?. Europace.

[29] Lee DC, Albert CM, Narula D, Kadish AH, Panicker GK, Wu E (2020). Estimating myocardial infarction size with a simple
electrocardiographic marker score. J Am Heart Assoc.

